# Lost and not found: randomized controlled trial of cognitive behavioral therapy for weight-loss in patients with chronic kidney disease

**DOI:** 10.1007/s10865-025-00583-w

**Published:** 2025-06-19

**Authors:** Katja Kurnik Mesarič, Jana Kodrič, Špela Bogataj, Andreja Marn Pernat, Aljoša Kuzmanovski, Bernarda Logar Zakrajšek, Jernej Pajek

**Affiliations:** 1 Department of Nephrology, Division of Internal Medicine, University Medical Centre Ljubljana, Ljubljana, Slovenia; 2 Department of Psychology, Faculty of Arts, University of Ljubljana, Ljubljana, Slovenia; 3 Child Psychiatry Unit, Division of Paediatrics, University Medical Centre Ljubljana, Ljubljana, Slovenia; 4 Faculty of sport, University of Ljubljana, Ljubljana, Slovenia; 5 Faculty of Medicine, University of Ljubljana, Ljubljana, Slovenia; 6 Dietetics and nutrition service, University Medical Centre Ljubljana, Ljubljana, Slovenia; 7 Management, Division of Surgery, University Medical Centre Ljubljana, Ljubljana, Slovenia

**Keywords:** Cognitive behavioral therapy, Chronic kidney disease, Obesity, Weight loss, Weight management

## Abstract

Introduction: Managing obesity in patients with chronic kidney disease is crucial for managing disease progression. Psychological interventions, particularly cognitive behavioral therapy, can support lifestyle changes. This study aimed to evaluate the efficacy of a cognitive behavioral therapy intervention for obesity management in patients with chronic kidney disease. Methods: Forty patients with chronic kidney disease (stages 2–4) were randomized to either an intervention group (nutritional and physical activity counseling and 16-week cognitive behavioral therapy) or a control group (nutritional and physical activity counseling only). Primary outcomes were body mass index (BMI) and proteinuria. Results: The intervention group lost an average of 5.42 kg (BMI decrease: 1.82 kg/m²), compared to 1.53 kg (BMI decrease: 0.53 kg/m²) in the control group. A significant group-by-time interaction was observed for BMI (F(1,36) = 32.24, *p* = 0.004, ŋ²=0.21), favoring the intervention group. Effects remained significant at three-month follow-up, with an average weight loss of 4.63 kg (BMI decrease: 1.59 kg/m²) in the intervention group and 2.51 kg (BMI decrease: 0.87 kg/m²) in control group (F(2,70) = 5.54, *p* = 0.026, ŋ²=0.12). Changes in proteinuria did not differ between groups. Conclusion: Cognitive behavioral therapy was effective and well-tolerated for promoting weight loss with most of the lost weight maintained at the three-month follow-up. This intervention may offer a valuable non-pharmacological treatment option for weight management in patients with chronic kidney disease.

## Introduction

Chronic kidney disease is one of the most prevalent chronic noncommunicable diseases, affecting approximatley 10% of global population (Kovesdy, 2022). Progression of the chronic kidney disease can lead to kidney failure, premature mortality, disability, reduced quality of life, psychosocial stress and increased economic burden. It is also closely related with other chronic noncommunicable diseases such as diabetes, hypertension and cardiovascular diseases (Francis et al., 2024). Obesity and its associated lifestyle factors (sedentary lifestyle, unhealthy diet, physical inactivity etc.) can play a significant role in development and progression of chronic kidney disease (Cisneros-García et al., 2023; Hannan et al., 2021; Mallamaci & Tripepi, 2024; Navaneethan et al., 2009; Nawaz et al., 2023; Wang et al., 2008). Obesity is related with increased blood pressure, inflammation, insulin resistance, metabolic disorders and hyperfiltration in kidneys, which all can accelerate kidney damage (Hall et al., 2021). utrition and physical activity play key roles in managing these risk factors, since balanced diet can reduce harmful substances that strain the kidneys, while regular physical activity helps control blood pressure, improve insulin sensitivity, and reduce inflammation (Schrauben et al., 2021; Sahu et al., 2024). Proteinuria (the presence of excess protein in the urine) is a key indicator of impaired kidney function and it goes together with glomerular hyperfiltration as seen in obesity and other glomerular disorders. It not only reflects existing damage but also contributes to further kidney injury and is strongly associated with faster progression of chronic kidney disease (Cravedi & Remuzzi, 2013). Proteinuria may improve after weight loss because losing weight helps to reduce hyperfiltration, reduce inflammation and improve insulin sensitivity (Praga & Morales, 2006).

Clinical guidelines for chronic kidney disease management recommend achieving a healthy weight and promoting healthy lifestyle as key steps to minimise the risk of progression to kidney failure (KDIGO Executive Committee, 2024). Traditionally, patients with chronic kidney disease have been offered dietary interventions, physical activity programs or surgical treatment (Conley et al., 2021; Navaneethan et al., 2009). However, lifestyle interventions are limited in terms of clinical effectiveness (Conley et al., 2021; Navaneethan et al., 2009) and often face challenges such as high dropout rates (diet and exercise interventions), limited availability (e.g. bariatric surgery) and poor long-term weight maintenance (Dalle Grave et al., 2018). In recent years, pharmacological approaches to manage obesity have gained wide acceptance, with sodium-glucose cotransporter-2 (SGLT2) inhibitors and glucagon-like peptide-1 (GLP-1) receptor agonists and dual GIP/GLP-1 receptor agonists demonstrating effectiveness in weight loss in patients with obesity and/or type 2 diabetes (Iqbal et al., 2023; Ma et al., 2023; Melson et al., 2024). However, their safety, applicability, and impact on weight management in patients with chronic kidney disease require further investigation (Taber-Hight et al., 2024). Also, this pharmacological treatment needs to be prescribed indefinitely to maintain effectiveness as rebound effects after discontinuation of these agents was demonstrated (surmount-4 trial, step-1 extension trial) (Aronne et al., 2024; Wilding et al., 2022).

Incorporating psychological approaches into the treatment of overweight and obese patients can significantly improve the effectiveness of lifestyle changes and increase motivation for weight loss (Castelnuovo et al., 2017). Cognitive factors, in particular, play an important role in eating behaviors (Jansen et al., 2015). Cognitive behavioral therapy is effective way for weight loss and management in general populations (Comșa et al., 2020; Jacob et al., 2018; Kurnik Mesarič et al., 2023b). Up to this point, there were no studies on cognitive behavioral therapy for parents with chronic kidney disease, with exception of one recent pilot study, examining the effects of telehealth-delivered cognitive behavioral and dietary intervention for weight loss in adult kidney transplantation recipients (Barchfeld et al., 2023). In that study, 32% of the participants in the intervention group achieved a 5% reduction in body weight, compared to 16.7% in the control group. The between-group difference was not statistically significant and the effect size was small (Barchfeld et al., 2023).

The efficacy of psychological interventions for obesity management in patients with chronic kidney disease remains understudied. The aim of our study was to evaluate the efficacy of the cognitive behavioral intervention for obesity management in patients with chronic kidney disease. We hypothesized that, when added to traditional approaches, the intervention would lead to greater reductions in excess body weight and proteinuria levels in patients with chronic kidney disease.

## Materials and methods

### Study design

The study was a randomized, controlled, open-label trial was designed to evaluate the efficacy of cognitive behavioral intervention on weight loss in patients with chronic kidney disease from stage 2 to 4. Participants were recruited at the outpatient nephrology clinic of University Medical Center Ljubljana, Slovenia. To be eligible for the study, patients had to meet the following criteria: (1) diagnosis of chronic kidney disease stage 2 to 4; and (2) body mass index above 30 kg/m^2^ or body mass index above 28 kg/m^2^ and waist circumference above 94 cm for men or 82 cm for women; (3) estimated daily proteinuria above 0.2 mg per day per g of creatinine in the urine and (4) age from 18 to 75 years. Type 2 diabetes was not an exclusion criterion. Patients were not included in cases with: (1) dementia; (2) acute psychiatric illness episode or poorly managed psychiatric illness; (3) bioimpedance findings of low lean body mass index below that expected for age and sex (or presence of any other sarcopenic obesity criteria); (4) active chronic inflammatory disease or active cancer; (5) active nephrotic syndrome; (6) heart failure (rating 3–4 on the New York Heart Association scale); (7) receiving induction immunosuppression therapy for autoimmune renal disease; (8) pharmacological treatment of obesity or any other active obesity treatment; (9) spontaneous weight loss of 5% or more in the last 6-month period or (10) any other clinical factor that puts the patient at risk with regard to metabolic stability and daily energy expenditure.

Initiation of pharmacological weight loss treatment was not permitted during the study period, and patients prescribed such treatment were excluded. Likewise, changes to the therapies affecting proteinuria (such as RAS inhibitors or SGLT2i) were not allowed during the study period. The study strictly adhered to the ethical principles of the Declaration of Helsinki and was approved by the National Medical Ethics Committee of the Republic of Slovenia (Document No. 0120 − 26/2023/3). Before enrolment in the study, all participants provided written informed consent. The clinical trial was registered at ClinicalTrials.gov (Identifier NCT05927337).

### Participants

**Fig. 1:**
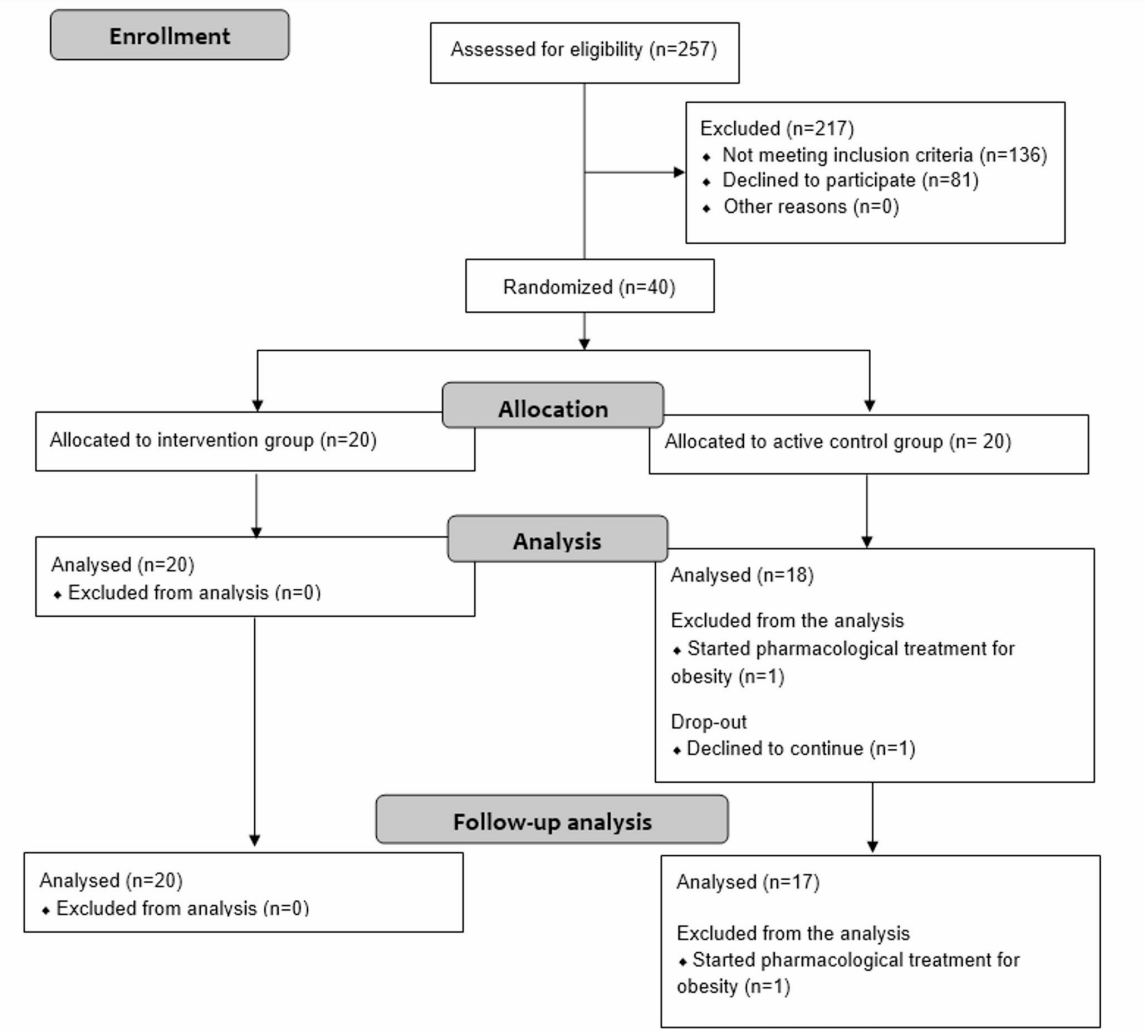
Study flow diagram

The flow of the study is presented in Fig. 1. Out of the 257 individuals initially assessed for eligibility, 40 were randomly assigned to either the intervention or active control group. In the control group, two patients were lost to all final assessments; one initiated pharmacological treatment for obesity and the second refused to continue participating. Baseline characteristics of included patients are presented in Table 1. No significant differences were found between the groups at baseline.

**Table 1:** Baseline characteristics divided by groups

	Intervention group			Control group				
	*M* (*SD*)	*Min*	*Max*	*M* (*SD*)	*Min*	*Max*	*t* ^*1*^ */U* ^*2*^	*p*
BMI (kg/m^2^)	36.65 (5.44)	28.0	53.3	33.7 (3.70)	28.0	42.6	2.01^1^	0.052
Proteinuria (mg/g)	926.0 (984.79)	201.4	3799.1	1472.1 (1716.98)	202.1	5625.7	168.50^2^	0.398
eGFR (ml/min/1.73m^2^)	46.4 (24.45)	15.0	88.0	47.1 (25.68)	15.0	89.0	192.40^2^	0.841
Body Weight (kg)	111.4 (17.66)	77.0	150.5	102.6 (15.21)	78.0	136.0	1.69^1^	0.098
Waist Circumference (cm)	115.8 (9.56)	103	143	112.6 (12.50)	90	136	0.92^1^	0.361
Cholesterol (mmol/L)	4.00 (1.03)	2.0	5.8	4.47 (1.03)	2.8	6.8	-1.46^1^	0.152
LDL Cholesterol (mmol/L)	2.42 (0.91)	0.6	4.1	2.63 (1.08)	1.4	4.5	195.50^2^	0.904
Systolic BP	152.6 (18.4)	115	198	150.1 (22.6)	112	190	0.38^1^	0.709
Diastolic BP	89.2 (13.8)	62	112	84.5 (12.6)	62	109	1.12^1^	0.272
CKD stage (number of patients) Stage 2 Stage 3 Stage 4	7 6 7			7 5 8				

IG = intervention group; CG = control group; BMI = body mass index; eGFR = estimated glomerular filtration rate; LDL = low-density lipoproteins; BP = blood pressure, CKD = chronic kidney disease, 1 = t-test, 2 = Mann-Whitney U test

Eleven patients were female (27.5%) and 29 were male (72.5%), with a mean age of 58.45 years (± 9.96). Nineteen (47.5%) were employed, 19 (47.5%) were retired and 2 (5%) were unemployed. The mean duration of chronic kidney disease was 7.35 years (± 5.67). Additionally, 34 patients (85%) had arterial hypertension, 14 (35%) had type 2 diabetes and 2 (5%) had psychiatric disorders (bipolar affective disorder and schizophrenia), both in remission.

### Outcome measures

Outcome measures were assessed at baseline and following the 16-week intervention. The primary endpoints were body mass index (BMI) and proteinuria. The secondary outcomes included additional measures of metabolic regulation (body weight, waist circumference, total cholesterol, and low-density lipoprotein (LDL) cholesterol levels) and indicators of kidney functioning (estimated glomerular filtration rate (eGFR), systolic and diastolic blood pressure). These were selected based on their clinical relevance in the context of chronic kidney disease and weight loss related interventions.

BMI was calculated based on body height and weight using the formula BMI = kg/m². Body mass index was also measured 3-months after the end of an intervention. Proteinuria was selected as the primary outcome because it is one of the most well-established surrogate endpoints in nephrology. Given the study’s sample size and duration, assessing hard endpoints such as mortality or progression to kidney failure was not feasible. Proteinuria was quantified from urine samples collected at baseline and post-intervention. Standard laboratory procedures were used, and proteinuria was reported in units of milligrams per gram of creatinine.

Body weight was recorded using a calibrated medical scale (accuracy: 0.1 kg). Waist circumference was measured in centimetres with a standardized measuring tape. Serum total cholesterol and LDL cholesterol levels were measured in mmol/L from blood samples using standard laboratory methods. Systolic and diastolic blood pressure were measured in the right arm while the patient was seated and at 5-minute rest, by a nurse using an adjustable cuff electronic sphygmomanometer.

### Study procedures

All eligible patients who consented to participate were assessed and randomly assigned in 1:1 ratio to the control or intervention group using gender stratification. Randomization was performed using the Clinical Trial Randomization Tool program form The National Cancer Institute’s Division of Cancer Prevention (https://ctrandomization.cancer.gov/tool). Detailed procedures are available in the study protocol (Kurnik Mesarič et al., 2023a, b).

The intervention group received a 16-week (12 session) individual program of cognitive behavioral therapy for obesity management, delivered by a licenced psychologist trained in cognitive behavioral therapy. The intervention was developed by a team of psychologists, medical doctors and researchers based on as based on *Individualised cognitive behavioral therapy for managing obesity* program (Dalle Grave et al., 2018), but it was shortened and adapted. Our intervention included two sessions of nutritional counselling, during which patients received explanations about the impact of food choices on the progression of chronic kidney disease (e.g., excessive intake of salt and fat, or frequent consumption of processed foods), as well as guidance tailored to the specific dietary needs associated with chronic kidney disease. In addition, two sessions of physical activity counselling were included, aimed at promoting regular exercise and addressing the specific physical limitations and recommendations relevant to individuals with chronic kidney disease. These sessions were scheduled on the days of initial and final assessments. The cognitive behavioral intervention program retained six core modules from the original (Dalle Grave et al., 2018): monitoring food intake, physical activity and body weight; changing eating; developing an active lifestyle; addressing obstacles to weight loss; addressing weight loss satisfaction; addressing obstacles to weight maintenance. Sessions were held weekly for the first eight weeks and biweekly for the last eight. A full description of the program is available in the study protocol or upon request (Kurnik Mesarič et al., 2023a, b).

The active control group received two sessions of individual nutritional counselling, delivered by dietitian and two sessions of individual physical activity counselling, delivered by kinesiologist, matching the content and timing of the intervention group’s nutrition and activity sessions. Active control group intervention was determined based on the existing standard clinical practice in our centre for chronic kidney disease patients with obesity that need body weight reduction (typically at least one session with a dietitian focusing on nutrition and exercise advice.).

### Data analysis

The sample size was calculated using G*Power software, version 3.1 (Faul et al., 2009), based on the results of the study by Fernandez-Ruiz et al. (2018), calculated on the size of the effect of weight reduction and the expected variability of this parameter. The alpha error probability was set to 0.05, the 1-beta error probability to 0.80, while the effect size (0.56) was taken from the previously mentioned study (Fernández-Ruiz et al., 2018). A sample size of 34 participants was calculated. Considering an expected drop-out rate of 20%, a total number of 40 participants was required to ensure adequate statistical power to detect a difference between the two study groups, with 20 participants assigned to each group.

Statistical analysis was carried out using IBM SPSS statistical software version 29 (IBM Corporation, USA). Normality distribution of variables was assessed using Kolmogorov-Smirnov test. Baseline group differences were assessed using the *t*-test for normally distributed variables and the Mann–Whitney *U*-test for variables that were not normally distributed. A repeated-measures ANOVA was conducted to evaluate the effects of group (intervention vs. control) as the between-subjects factor and time (pre- and post-intervention) as the within-subjects factor on BMI, body weight, waist circumference, and total cholesterol. Since the assumption of sphericity was violated, the Greenhouse-Geisser correction was applied to adjust the results. A paired-samples t-test was used to determine within-group differences over time for BMI.

Due to violations of assumptions, Friedman ANOVA was conducted to assess changes in estimated daily proteinuria, LDL cholesterol, systolic and diastolic blood pressure across the two time points (pre-intervention, post-intervention) for the intervention and control groups. Man-Whitney U test was used to determine within-group differences over time for proteinuria variable, due to non-normal distribution.

The study employed an intention-to-treat (ITT) analysis as the primary method of analysis. Cohen’s d effect size (ES) was used to assess the observed differences magnitude for each group. A magnitude of 0.2 < ES ≤ 0.5 was considered small, while a magnitude of 0.5 < ES ≤ 0.8 and ES > 0.8 was treated as moderate and large (Cohen, 2013).

## Results

### Tolerability and adherence

The intervention for patients with chronic kidney disease was generally well tolerated. Notably, all 20 participants randomized to the cognitive behavioral therapy condition completed the full duration of the program, indicating strong retention in a population often characterized by high treatment burden and frequent comorbidities. Adherence to sessions was high, 11 participants attended all 12 sessions, 2 attended 11 sessions, 4 attended 10 sessions, 2 attended 9 sessions, and 1 attended 8 sessions. Missed sessions were primarily attributed to seasonal illnesses (e.g., colds) and work obligations. There was a total of 11 homework assignments for participants in intervention group, completion rate was from 41.7 to 83.3% (*M* = 69.2 ± 10.5). Adherence to nutritional counselling and physical activity counselling was 100% for all 38 patients (from both groups) who completed the program.

Subjective observations from the psychologist delivering the intervention indicated occasional challenges with participant motivation. Some individuals required additional encouragement to maintain engagement with the program. To address challenges with attending sessions, adjustments were made to the scheduling of sessions to accommodate participants’ needs, such as offering more flexible timings and reminders.

### Body mass index

On average, the intervention group experienced a BMI reduction of 1.82 kg/m^2^, while the control group showed an average reduction of 0.53 kg/m^2^. Descriptive statistics for each group at each time point are presented in Table 2. A repeated-measures ANOVA indicated a significant interaction effect between group (intervention vs. control) and time (pre- vs. post- intervention) on BMI (*F*(1,36) = 32.24, *p* = 0.004, *ŋ*2 = 0.21) favouring the intervention group.

**Table 2:** Pre- and post- intervention BMI values per group

Group	Baseline M (SD)	After intervention M (SD)	Pre- to post-change (95% CI)	*p*	ES (pre-post)
Intervention	36.64 (5.44)	34.83 (4.94)	1.82 (1.20 to 2.43)	**< 0.001**	-0.35
Control	33.14 (3.16)	32.61 (3.89)	0.53 (-0.7 to 1.14)	0.081	-0.15

CI = confidence interval; ES = effect size

Within-group analysis showed a significant pre–post reduction in BMI for the intervention group. Control group showed did not show a significant pre-post reduction in BMI.

A decrease in BMI was significantly associated with the percentage of homework completed by participants (*r* = 0.793, *p* < 0.001), but not with session attendance (*r* = 0.243, *p* = 0.302).

#### Three-month follow-up after the intervention

Three months after the program, the intervention group had an average BMI of 35.06 kg/m^2^ (± 5.14), while the control group had an average BMI of 32.28 kg/m^2^ (± 4.16). Compared to baseline, the intervention group experienced a significant BMI reduction of 1.59 kg/m^2^ (*p* < 0.001), whereas the control group showed a significant reduction of 0.87 kg/m^2^ (*p* = 0,037). The interaction effect remained significant at follow-up, again favouring the intervention group (*F*(2,70) = 5.54, *p* = 0.026, *ŋ*2 = 0.12). Descriptive statistics for each group at each time point are presented in Table 3.

**Table 3:** Pre-intervention and follow-up BMI values per group

Group	Baseline M (SD)	Follow-up M (SD)	Pre- to follow-up change (95% CI)	*p*	ES (pre-post)
Intervention	36.64 (5.44)	35.06 (5.14)	1.58 (0.82 to 2.34)	**< 0.001**	-0.30
Control	33.14 (3.16)	32.28 (4.16)	0.87 (0.06 to 1.95)	**0.037**	-0.23

CI = confidence interval; ES = effect size

**Fig. 2:**
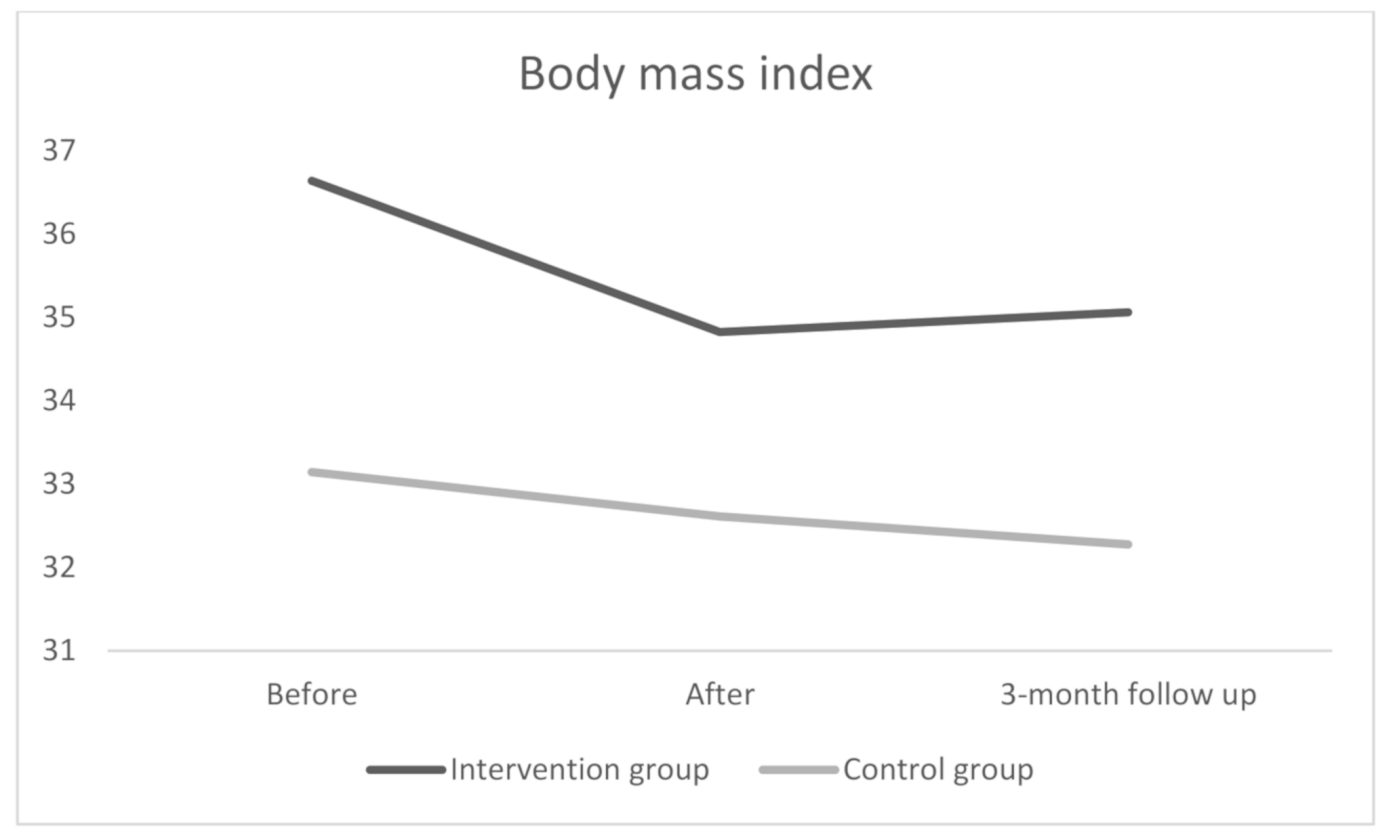
Body mass index change

From post intervention to follow up, the intervention group showed an increase BMI of 0.24 kg/m^2^, while the control group experienced a decrease of 0.33 kg/m^2^. However, these changes were not statistically significant in either group. Figure 2 presents the average BMI over time for both groups.

### Proteinuria

Intervention group decreased proteinuria on average for 113.14 mg/g, and control group decreased proteinuria on average for 133.06 mg/g. Descriptive statistics for each group at both time points are shown in Table 4. Friedman ANOVA in the intervention group revealed a significant effect of time. In the control group, no significant effect of time was observed. A Mann-Whitney U test was conducted to compare the change in estimated daily proteinuria between the intervention and control group. The difference in proteinuria change scores between groups was not statistically significant (*U* = 175.00, *p* = 0.897).

**Table 4:** Pre- and post- intervention proteinuria values per group

Group	Baseline M (SD)	After intervention M (SD)	Pre- to post-change (95% CI)	χ2	*p*	ES (pre-post)
Intervention	927.00 (984.79)	813.86 (1005.99)	113.14 (-142.50 to 386.66)	5.00	**0.025**	-0,11
Control	1566.28 (1785.62)	1433.22 (1820.01)	133.06 (-183.39 to 449.54)	2.00	0.157	-0.07

CI = confidence interval; ES = effect size

The Pearson correlation between changes in BMI and proteinuria across the entire sample was *r* = 0.06, *p* = 0.744, indicating no significant association.

### Secondary outcomes

Over the 16-week intervention, 11 out of 20 patients in the intervention group achieved more than 5% weight loss, and 1 of those patients achieved more than 10% weight loss. In contrast, in the control groups, 3 out of 20 patients achieved more than 5% weight loss, with no patients reaching more than 10% weight loss. A chi-square test of independence was conducted to examine whether there was an association between group membership (intervention vs. control) and the achievement of the 5% weight loss (achieved vs. not achieved). The results showed a significant association between group membership and achievement of 5% weight loss *χ*^2^(1,*N* = 38) = 5.98,*p* = 0.014. The intervention group lost on average 4.82% body weight, while the control group lost on average 1.83% body weight.

On average, the intervention group lost 5.42 kg (± 3.91), while the control group lost 1.53 kg (± 3.65). A repeated-measures ANOVA indicated a significant interaction effect between group (intervention vs. control) and time (pre- vs. post- intervention) on body weight (*F*(1,36) = 9.47, *p* = 0.004, *ŋ*2 = 0.20), favouring the intervention group. On a weekly basis, the intervention group lost an average of 340 g, whereas the control group lost 96 g.

From baseline to follow-up intervention group lost on average 4.63 kg (± 4.58), compared to 2.41 kg (± 4.42) in the control group. The interaction effect between group and time remained significant at follow up (*F*(2,70) = 4.15, *p* = 0.033, *ŋ*2 = 0.11), again favouring the intervention group.

In terms of waist circumference, the intervention group lost an average of 4.9 cm (± 2.9), while the control group lost 1.8 cm (± 3.9). A repeated-measures ANOVA also indicated a significant interaction effect for waist circumference results (*F*(1,36) = 7.82, *p* = 0.008, *ŋ*2 = 0.18).

For total cholesterol, the intervention group showed an average decrease of 0.04 mmol/L (± 0.54), while the control group showed no change (0.00 mmol/L ± 0.79). Repeated measures ANOVA showed no significant interaction effects (*F*(1,36) = 0.30, *p* = 0.587, *ŋ*2 = 0.01). For LDL cholesterol, the intervention group had an average decrease of 0.13 mmol/L (± 0.29), while the control group decreased by 0.09 mmol/L (± 1.02). Friedman ANOVA did not reveal significant effect of time in any groups for LDL cholesterol change (intervention group: *χ2*(1) = 1.47, *p* = 0.225; control group *χ2*(1) = 0.82, *p* = 0.366).

For systolic blood pressure, the intervention group decreased by 8.88 mmHg (± 18.91), while the control group decreased by 4.31 mmHg (± 16.26). However Friedman ANOVA did not reveal significant effect of time in any groups for systolic blood pressure change (intervention group: *χ2*(1) = 1.47, *p* = 0.225; control group *χ2*(1) = 0.69, *p* = 0.405). For diastolic blood pressure, the intervention group decreased by 2.18 mmHg (± 10.93), while the control group decreased by 0.38 mmHg (± 1.16). Friedman ANOVA did not reveal significant effect of time in any groups for blood pressure (intervention group: *χ2*(1) = 0.25, *p* = 0.617; control group *χ2*(1) = 1.92, *p* = 0.166).

### Adverse events

No adverse events were observed during the intervention. Specifically, there were no urgent visits to the nephrology outpatient clinic, instances of acute kidney failure, episodes of hypotension, or disturbances in consciousness.

## Discussion

This study is the first to examine the efficacy of a cognitive behavioral intervention for obesity management in patients with chronic kidney disease. By focusing on this population, we aimed to provide insights that may help slow disease progression and reduce obesity-related risks. The intervention was multidisciplinary and emphasized non-pharmacological strategies, with cognitive behavioral therapy as a key component. The practical feasibility of cognitive behavioral therapy for weight management adds to its clinical relevance and highlights its potential for broader implementation. Our intervention significantly reduced body mass index and body weight compared to the control group. However, there were no statistically significant differences between groups in proteinuria, cholesterol levels or blood pressure, likely due to the modest weight loss and small sample size.

Our hypothesis regarding BMI reduction was supported. Participants in the intervention group achieved a significant average BMI reduction of 1.29 kg/m². Although this is somewhat lower than reductions reported in meta-analyses of lifestyle interventions in patients with chronic kidney disease (Conley et al., 2021; Navaneethan et al., 2009), it remains statistically significant. These meta-analyses included interventions of varying durations (3 to 24 months), which may explain the discrepancy. A recent pilot study evaluating cognitive behavioral and dietary intervention for weight loss in kidney transplant recipients reported an average BMI reduction of 0.9 in the intervention group compared to 0.2 in the control group over a 12-session, six-month program (Barchfeld et al., 2023). In our study, participants in both groups achieved larger BMI reductions. At the three-month follow-up, participants in the intervention group retained most of the weight loss and the reduction of BMI compared to baseline remained statistically significant. Our findings are in line with previous research demonstrating the efficacy of cognitive behavioral therapy for weight loss and maintenance in both the general population and in individuals with type 2 diabetes (Comșa et al., 2020; Kurnik Mesarič et al., 2023b). These studies support the use of cognitive behavioral therapy based interventions in populations with chronic conditions, which is particularly relevant given the comorbidities often present in patients with chronic kidney disease. In our study, 11 participants in the intervention group, compared to only 3 in the control group, achieved clinically meaningful weight loss (≥ 5%).

The average weight loss difference between intervention and control groups in our study was 3.9 kg, aligning closely with meta-analytic findings (Conley et al., 2021), although comparisons are complicated by varied intervention durations. Results from other meta-analysis are showing, that cognitive behavioral therapy is effective for weight loss in general population (Jacob et al., 2018), where the average difference in lost weight among intervention and control groups was 1.7 kg in favour of intervention groups.

The amount of weight lost was significantly associated with homework completion in the intervention group, but not with session attendance. This suggests that participants who were more actively engaged between sessions lost more weight than those who attended sessions but were less active outside of them. It appears that that active participation between sessions was one of the key drivers of behavior change, and that aligns with previous research suggesting that self-regulation skills, self-efficacy and autonomous motivation are best predictors for beneficial weight loss outcomes (Teixeira et al., 2015).

The intervention group received more treatment hours (16 vs. 4) and had higher treatment frequency compared to control group. While the control group had only two visits of nutritional and exercise counselling (one at inclusion and another at 16 weeks), the intervention group had weekly sessions for the first eight weeks, followed by bi-weekly sessions for the next eight weeks. Given that exposure time was not equal between groups, this difference should be considered when interpreting the results. Exposure time is particularly important in behavioural research (Voils et al., 2012).

The fact that all participants in the intervention group remained in the study, with high attendance and homework completion, suggests that the intervention was both acceptable and feasible for individuals with chronic kidney disease. This is particularly notable given the multiple medical and psychosocial challenges faced by this population.

While initial weight loss outcomes were promising, long-term maintenance remains a significant challenge. Key strategies for successful weight maintenance include maintaining a regular eating schedule with breakfast, engaging in consistent physical activity, choosing low-fat and low-calorie foods, and regularly monitoring body weight (Dalle Grave et al., 2018). However, several factors may increase the risk of relapse in patients with chronic kidney disease, such as the burden of comorbid conditions that limit physical activity, strict dietary restrictions, and persistent fatigue. A more in-depth understanding and targeted management of these barriers will be essential for the development of future interventions.

Our hypothesis on estimated daily proteinuria reduction was not supported by our results. The between-group comparison did not reveal a significant difference and overall, changes were small. There was a non-significant imbalance in baseline proteinuria, with greater variability in the control group. Furthermore, no significant changes in blood pressure were observed in either group, which may have limited the potential for proteinuria reduction. In meta-analysis of four studies for weight loss in patients with chronic kidney disease and obesity (Conley et al., 2021), proteinuria reduction was greater in intervention groups for 0.29 g/day, and the authors conclude that moderate weight loss might not induce proteinuria reduction. In our study, we used spot urine samples (not 24-hours collections), and that may have influenced results, especially for patients with more than 3 g/day of proteinuria (Chen et al., 2019).

Waist circumference decreased by an average of 3.2 cm more in the intervention group—a larger difference than reported in meta-analyses (0.68 cm), though prior studies were few and of low quality (Conley et al., 2021). No significant changes were observed in total or LDL cholesterol, consistent with other lifestyle intervention studies.

Some participants in the control group achieved similar weight loss to those in the intervention group, while some individuals in the intervention group did not experience any weight loss, despite receiving cognitive behavioral therapy. This could be attributed to different factors, such as individual motivation, which is crucial in lifestyle change and weight loss success (Cheng et al., 2023; Comșa et al., 2020; Lanoye et al., 2019; Teixeira et al., 2012a; Teixeira, Silva, Teixeira et al., 2012a, b). Behaviors essential for successful lifestyle changes are linked to long-term positive outcomes and benefits. However, immediate rewards and positive consequences often stem from unhealthy habits, making it challenging to overcome unhealthy behavioral patterns (Michaelsen & Esch, 2023; Zhang et al., 2021). As such, motivation could be one of the key factors influencing the success of this intervention. The finding that some patients with less intensive intervention performed well, while others with more extensive intervention still struggled, highlights the complexity of individual weight loss journeys. These results suggest that a one-size-fits-all approach may not be sufficient and highlights the potential need for stepped-care interventions in weight management, where some patients may benefit from brief interventions while others may require more intensive, multidisciplinary approaches. The stepped-care model has demonstrated efficacy and cost-effectiveness in mental health area (Ho et al., 2016), and future protocols should consider including motivation assessments to better tailor interventions to individual needs.

### Strengths and limitations

The study employed a multidisciplinary, non-pharmacological approach, with cognitive behavioral therapy as core. The use of cognitive behavioral therapy for weight management in clinical practice enhances study’s practical relevance and highlights its potential for broader implementation. Notably, there were no dropouts in the intervention group. However, the small sample size limits the generalizability of our findings, especially those related to proteinuria. As sample size was determined based on the body mass index outcome, the study lacked sufficient power to detect significant differences in proteinuria, as indicated by the post-hoc analysis. Additionally, the intervention’s nature prevented blinding of investigators and participants. With a duration of 16 weeks and an average weight-loss goal of 0.5 kg per week, the total weight loss was limited, and a modest absolute loss was achieved. A longer intervention period may yield a more substantial result.

### Future directions

Future research should continue to focus on proteinuria as a primary outcome and consider calculating sample size based on expected changes in proteinuria. A larger sample may yield different results, and more precise inclusion criteria related to baseline proteinuria levels could enhance the interpretability of findings.

Incorporating motivational interviewing techniques into the intervention may improve efficacy of the program. Extending the intervention duration and evaluating its success in group settings would also be worthwhile, as group formats could improve both weight-loss outcomes and cost-effectiveness. Studies show that both, individual and group cognitive behavioral therapy for managing obesity are similarly effective (Cresci et al., 2007; Ricca et al., 2010).

Additionally, studying the effectiveness of remote (online) or combined versus in-person interventions could provide valuable insights into how delivery formats impact patient engagement, outcomes, and accessibility. With the increasing availability of pharmacological treatments for managing obesity, we strongly propose to incorporate cognitive behavioral therapy for lifestyle modifications alongside these treatments.

## Conclusion

In summary, our study demonstrated the efficacy of cognitive behavioral therapy for obesity management in patients with chronic kidney disease in comparison to control group, with no psychological support. The intervention was well tolerated by patients. These findings suggest that the intervention might serve as an effective non-pharmacological tool to manage obesity in patients with chronic kidney disease.

## Data Availability

The datasets generated during and/or analysed during the current study are available from the corresponding author on reasonable request.
